# How meteorological factors impacting on scrub typhus incidences in the main epidemic areas of 10 provinces, China, 2006–2018

**DOI:** 10.3389/fpubh.2022.992555

**Published:** 2022-10-19

**Authors:** Yizhe Luo, Longyao Zhang, Heng Lv, Changqiang Zhu, Lele Ai, Yong Qi, Na Yue, Lingling Zhang, Jiahong Wu, Weilong Tan

**Affiliations:** ^1^Department of Epidemiology, School of Public Health, Nanjing Medical University, Nanjing, China; ^2^Nanjing Bioengineering (Gene) Technology Centre for Medicine, Nanjing, China; ^3^Department of Biostatistics, School of Public Health, Nanjing Medical University, Nanjing, China; ^4^College of Life Science, Fujian Agriculture and Forestry University, Fuzhou, China; ^5^Guizhou Medical University, School of Basic Medical Sciences, Guiyang, China

**Keywords:** meteorological factors, scrub typhus, risk, distributed lag non-linear models, lag effect

## Abstract

Scrub typhus, caused by *Orientia tsutsugamushi*, is a serious public health problem in the Asia-Pacific region, threatening the health of more than one billion people. China is one of the countries with the most serious disease burden of scrub typhus. Previous epidemiological evidence indicated that meteorological factors may affect the incidence of scrub typhus, but there was limited evidence for the correlation between local natural environment factors dominated by meteorological factors and scrub typhus. This study aimed to evaluate the correlation between monthly scrub typhus incidence and meteorological factors in areas with high scrub typhus prevalence using a distributed lag non-linear model (DLNM). The monthly data on scrub typhus cases in ten provinces from 2006 to 2018 and meteorological parameters were obtained from the Public Health Science Data Center and the National Meteorological Data Sharing Center. The results of the single-variable and multiple-variable models showed a non-linear relationship between incidence and meteorological factors of mean temperature (Tmean), rainfall (RF), sunshine hours (SH), and relative humidity (RH). Taking the median of meteorological factors as the reference value, the relative risks (RRs) of monthly Tmean at 0°C, RH at 46%, and RF at 800 mm were most significant, with RRs of 2.28 (95% CI: 0.95–5.43), 1.71 (95% CI: 1.39–2.09), and 3.33 (95% CI: 1.89–5.86). In conclusion, relatively high temperature, high humidity, and favorable rainfall were associated with an increased risk of scrub typhus.

## Introduction

Scrub typhus is an acute natural infectious disease caused by *Orientia tsutsugamushi* (1, 2). Historically, scrub typhus was only prevalent in the Asia-Pacific region, but with the development of tourism and trade between continents, local cases have also appeared in Europe, the Middle East, and Latin America (3). Globally, at least 12 million people were infected each year and it is probably the most common cause of non-malarial fever in the Asia-Pacific region (3). In recent years, the number of scrub typhus cases in China has continued to increase, and outbreaks have frequently occurred in some areas. The scope of new epidemic areas is gradually expanding, and millions of people are at risk of *Orientia tsutsugamushi* infection (4–7).

As an insect-borne disease, *Orientia tsutsugamushi* selects rodent hosts (*Apodemus agrarius and Suncus murinus*, etc.) as the main source of infection. Chigger larvae are the vectors, and are transmitted to humans through the bite of chigger mite larvae carrying *rickettsia* (8, 9). Typical clinical symptoms include high fever, toxemia, eschar, rash, and swollen lymph node (10).

Seasonal and geographic differences may affect the prevalence of scrub typhus by affecting the activity of scrub typhus vectors and hosts. Climate is related to the spread and prevalence of diseases, and global warming may provide opportunities for the spread of vector-borne diseases (11). For example, high RH, high Tmean, and abundant RF favor chigger reproduction, which provides excellent opportunities for the spread of chigger-related diseases. Furthermore, climate affects the risk of *tsutsugamushi* disease by affecting rodent populations, the proportion of rodents infected with pathogens, the number of chigger mites, and the frequency human exposure (12, 13).

Both chigger and host density are affected by environmental factors, many studies have proposed the relationship between natural environment factors and the incidence of scrub typhus, providing valuable information for us to understand the prevalence and spread of scrub typhus (such as Tmean, RH and RF) (5, 6). Through Poisson regression model, Wu et al. found that forest cover, Tmean and RF would increase the risk of scrub typhus (14). Yao et al. studied the risk factors of scrub typhus in northern China from 1980 to 2013 and found that abundant RF (over 400 mm), SH (140–180 h), suitable Tmean (9-14°C), farmland and high RH (62- 65%) significantly contributed to the spatial distribution of scrub typhus and the increased disease risk (15). Interestingly, in southern China, it was found to be more favorable for the spread of scrub typhus when the Tmean was more than 15°C and the RH was <63%. Due to different in research areas, methods, and the extent of data mining, these studies still have many limitations and cannot fully reveal and explain the reasons for the increasing incidence of scrub typhus in China.

Firstly, the relationship between scrub typhus and meteorological factors has not been fully extrapolated. Secondly, previous studies have rarely considered the lag effect of meteorological factors, and the time unit setting of the lag effect research is not detailed. Most of them selected some old models, and the results obtained are not reliable. Inconsistent results due to differences in models and regions, do not provide good predictive power, and cannot be used as a decision-making tool. A more suitable prediction model for scrub typhus has not been found. One of the key reasons is that changes in meteorological factors in different regions and degrees have different effects on the occurrence and development of diseases.

This study covered the epidemiological characteristics of scrub typhus from January, 2006 to December, 2018. On the basis of historical data, the DLNM was used to predict the relationship between meteorological factors and the incidence of scrub typhus in 10 provinces. To our knowledge, this is the largest epidemiological study of scrub typhus to date, covering the largest number of provinces and the longest period.

Humans generally lack immunity to scrub typhus and no effective vaccine is available. Therefore, exploring the relationship between climate change and scrub typhus will help the public health department to grasp the pattern of *tsutsugamushi* in multiple ways, take targeted prevention and control measures more effectively. We aimed to investigate the potential impact of various climatic variables on disease transmission opportunities in areas with high *tsutsugamushi* prevalence, taking into account the lag time. This will provide a new insight into the potential impact of climate change on scrub typhus transmission to help develop early warning systems.

## Materials and methods

### Data source and collection of case data

During the period from 2006 to 2018, monthly data of scrub typhus of 31 provinces in China, including the number of cases and incidence, were obtained from the National Notifiable Disease Report System. These data are available from the China Public Health Science Data Center (https://www.phsciencedata.cn/Share/en/index.jsp). Population data were obtained from the National Bureau of Statistics of the People's Republic of China (http://data.stats.gov.cn). All clinical diagnosis cases met the criteria for scrub typhus (http://www.chinacdc.cn) issued by the Chinese Center for Disease Control and Prevention in 2009.

### Meteorological data

The monthly meteorological data of each province from 2006 to 2018, including Tmean (°C), average RF (mm), average SH (h), average RH (%) were obtained from 272 sites enclosed in meteorological monitoring stations in China (http://data.cma.cn/wa) (Supplementary Figure 1).

### Statistical analysis

Although some provinces have large increasement in scrub typhus incidence, the actual incidence was very low, so we did not include them in the model. Finally, we selected top 10 average annual incidence provinces as study region in this study. In the descriptive analysis, mean, standard deviation, quartiles (*P*25, median, 75), minimum, and maximum were used to describe the distribution of incidence of scrub typhus and meteorological variables. Analysis of variance (ANOVA) test was applied to analyze the values of meteorological among the four seasons: spring (March to May), summer (June to August), autumn (September to November), and winter (December to February). A *Kruskal-Wails* test was used to examine the scrub typhus incidences among the four seasons. The significance level of all these analyses was set at 0.05 in two-tailed tests.

DLNMs, which can flexibly describe relationships and explore underlying lag non-linear effects, were used to uncover the association between meteorological and scrub typhus cases (16). In order to reduce the confounding effects and avoid collinearity in the DLNMs, three approaches were applied for analyzing the data. First, we selected the 10 provinces with the highest average annual incidence of scrub typhus as the research objects. Second, we used pairwise observations to compute the *Pearson*'s correlation coefficients between meteorological and scrub typhus incidence. Finally, we observed the effects of weather variables by single-variable and multiple-variable analysis, which can help identify the effects of other variables.

When fitting the DLNM, the relative risks at different values and lag months would be read out. The maximum number of lag months was determined by the smallest quasi-Bayesian information criterion (QBIC) in the multiple-variable DLNM. Finally, 7 months were set as the maximum number of lag months. Long-term cumulative risk referred to the cumulative 7-month risk.

In the single-variable model, in addition to the weather conditions, we further considered the factors of season, temporal trends, quantile groups for average incidence and previous month incidence.

The added variables in the multiple-variable model were used to control the significant confounders including Tmean, RH, RF, temporal trend, season factors, quantile groups for average incidence and previous month incidence.

Package “dlnm” (version 4.1.3, https://cran.r-project.org/web/package/dlnm/index.html) was used to specify the intersection of quadratic splines of three meteorological variables, predict and plot the results of the fitted model (contour plots, cumulative exposure-response curves, and 3-D plots) (17). DLNM was used to calculate the relative risk of scrub typhus under different meteorological factors with varying lag times (0–7 months).

We defined the cross-basis matrices when building DLNM. The cross-basis for weather factors was specified by B-spline using the function bs from the package SPLINES in R software (version 4.1.3, https://www.rdocumentation.org/packages/splines/versions/4.1.3). Regarding the space of lags, we evaluated the time lag from 0 to 7 months. The knots for the spline for lags were placed at equally spaced value on the log scale of lags, using the function lognots. The prediction values here were centered at the median of each meteorological variable.

### Sensitivity analysis

QBIC was used to select the optimal number and location of nodes reduced to determine natural splines. The final model should determine the minimum sum of QBICs for the ten selected provinces. AIC was used to select time-degree variables, including natural cubic splines of elapsed time, with one degree of freedom (df = 1) per year to control for long-term trends in meteorological factors across provinces. All statistical analyses were performed by two-tailed tests with a significance level of 0.05, using the Joinpoint Regression Program (version 4.9.0.0) developed by the National Cancer Institute (NCI), ArcGIS 10.2 (ESRI, Redlands, CA, USA) and R software (packages “dlnm”, “mgcv” and DescTools) (version 4.1.3, R Foundation for Statistical Computing, Vienna, Austria).

## Result

### Scrub typhus distribution in China from 2006 to 2018

**Figure 1:**
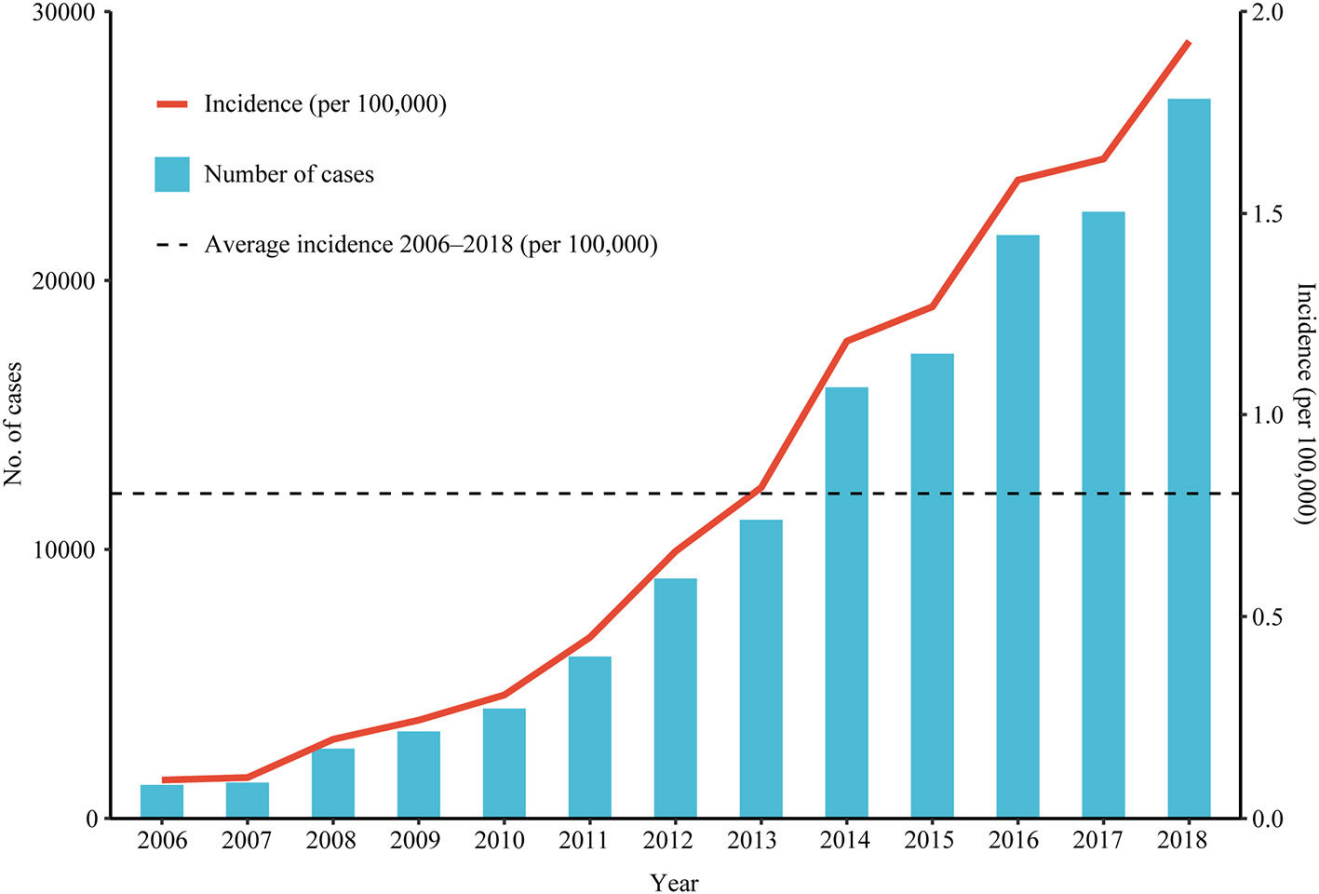
The incidence and number of scrub typhus cases reported in China from 2006 to 2018. Number of cases and incidence by year.

**Figure 2:**
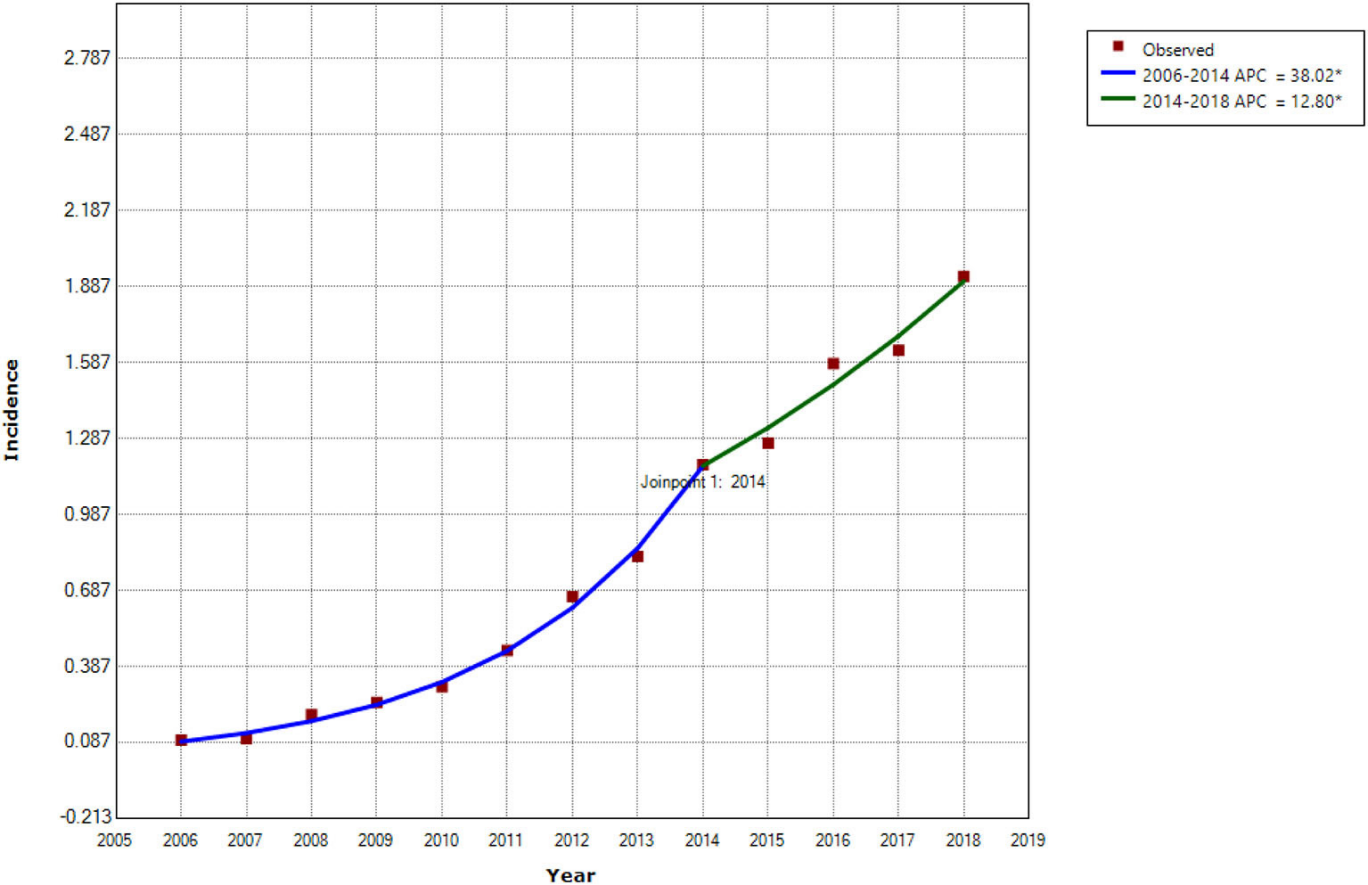
Trends in incidence of scrub typhus and joinpoints in China from 2006 to 2018. *Statistically significant trends. APC, Annual Percentage Change.

This study consisted of 142,849 scrub typhus cases between January 1, 2006 and December 31, 2018. The annual incidence showed a significant upward trend (Cochran-Armitage trend test Z = 280.82, *P* < 0.001), and the annualized average incidence was 0.641 per 100,000 (Figure 1). The national annual incidence of scrub typhus increased significantly from 0.095 per 100,000 in 2006 to 1.183 per 100,000 in 2014, with an APC of 38.02% (95% CI: 33.2–43.0%, *P* < 0.001), then increased to 1.93 per 100,000 in 2018, with an APC of 12.8% (95% CI: 1.8–25.0%, *P* = 0.027) (Table 1; Figure 2; Supplementary Figure 2). Among the 10 provinces with the highest incidence rates, Guangxi and Jiangxi had a larger increase in the incidence of scrub typhus with an AAPC of 53.3% (95% CI: 47.3–59.6%, *P* < 0.001) and 58.9% (95% CI: 45.0–74.1%, *P* < 0.001), respectively.

**Table 1:** The annual percentage changes (APC) and the joinpoint year range for scrub typhus in China from 2006 to 2018.

**Province**	**Joinpoint year range**	**APC, %**	***P-*values**
National	2006–2014	38.0* (33.2, 43.0)	<0.001
	2014–2018	12.8* (1.8, 25.0)	0.027
Anhui	2006–2018	20.7* (11.6, 30.7)	<0.001
Fujian	2006–2013	34.0* (22.8, 46.2)	<0.001
	2013–2018	8.6 (−6.1, 25.8)	0.228
Guangdong	2006–2013	37.7* (28.7, 47.3)	<0.001
	2013–2018	12.8* (0.8, 26.3)	0.039
Guangxi	2006–2014	61.5* (55.1, 68.1)	<0.001
	2014–2018	38.2* (23.0, 55.3)	<0.001
Hainan	2006–2018	38.6* (24.0, 55.0)	<0.001
Jiangsu	2006–2009	−15.4 (−58.6, 72.7)	0.573
	2009–2015	67.3* (21.6, 130.2)	0.009
	2015–2018	−22.4 (−62.0, 58.5)	0.404
Jiangxi	2006–2018	58.9* (45.0, 74.1)	<0.001
Shandong	2006–2018	16.0* (8.6, 23.8)	<0.001
Sichuan	2006–2018	29.1* (16.1, 43.5)	<0.001
Yunnan	2006–2018	31.0* (27.4, 34.7)	<0.001

^*^Statistically significant trends. APC, annual percentage change.

**Figure 3:**
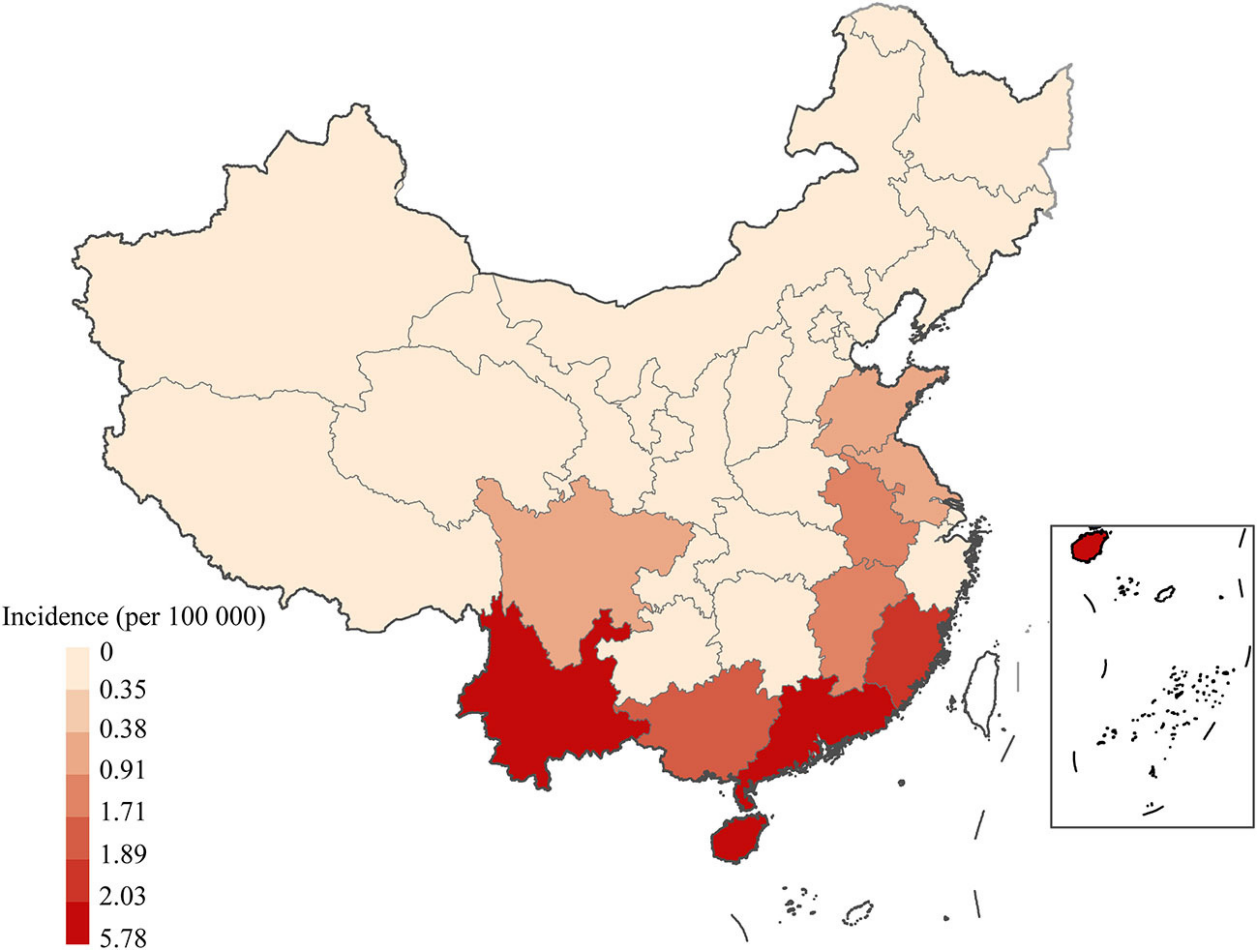
Spatiotemporal distribution of scrub typhus in China from 2006 to 2018. Annual incidence of scrub typhus per 100,000 people in the 31 Chinese provinces investigated.

From 2006 to 2018, scrub typhus predominantly circulated in the south, southeast and southwest of China (Figure 3). At least eight provinces in mainland China had more than 5,000 scrub typhus cases, including 38,439, 35,420, 12,198, 11,718, 9,935, 9,299, 8,642, and 5,509 in Guangdong, Yunnan, Anhui, Guangxi, Fujian, Jiangsu, Shandong and Jiangxi, respectively.

A heat map was used to show seasonal patterns, which displayed that the national incidence was lower in winter (December to February) and higher in autumn (September to November) (Supplementary Figure 3).

### Meteorological factors distribution in China from 2006 to 2018

**Figure 4:**
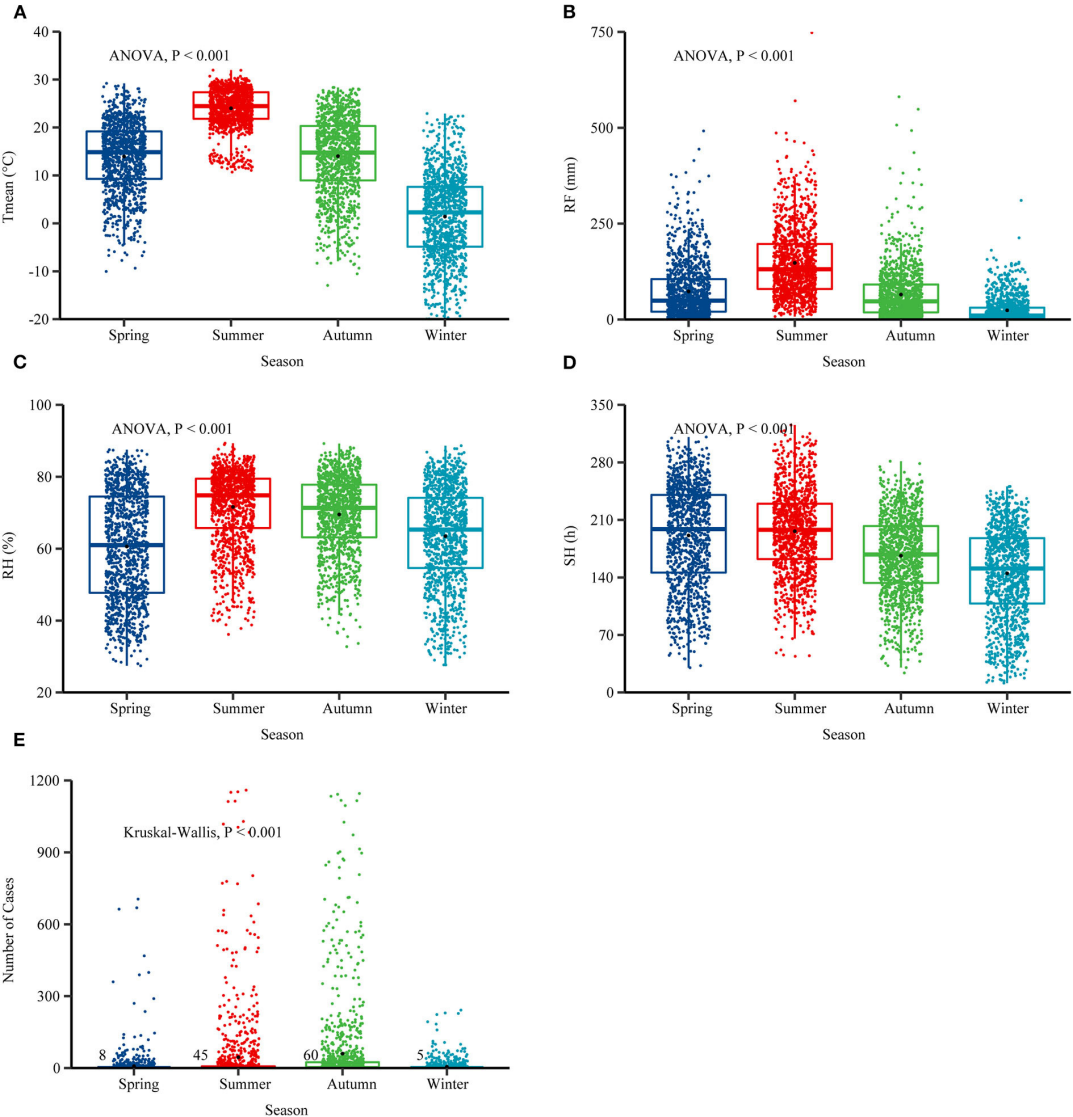
Boxplots of four meteorological conditions and the number of scrub typhus cases in four seasons from 2006 to 2018. **(A–D)** Seasonal patterns of weather conditions. **(E)** Seasonal patterns of scrub typhus. The analysis of variance (ANOVA) test was applied to test whether the values between the four seasons were statistically significant. The *Kruskal-Wallis* test was used to detect the cases of scrub typhus in four seasons, spring (March-May), summer (June-August), autumn (September-November) and winter (December-February). Mean temperature (Tmean), rainfall (RF), sunshine hours (SH), and relative humidity (RH).

The national monthly Tmean and average RH were 13.29°C and 66.30%. The RF was 77.63 mm, and the SH was 174.68 h (Table 2). In the selected 10 provinces, the average RH was 74.19%, the RF was 115.96 mm, and the SH was 155.94 h (Supplementary Table 1). The boxplots of meteorological conditions showed clear variations in the four seasons from 2006 to 2018 (Figures 4A–D), and the incidence of scrub typhus also showed seasonal variations, with the highest average number of cases in autumn (Figure 4E). The Tmean, RH, and RF were higher in summer. A similar pattern was also found in the selected 10 provinces (Supplementary Figure 4).

**Table 2:** Descriptive statistics for monthly scrub typhus cases and weather conditions in China from 2006 to 2018 (*n* = 4,836).

**Variables**	**Mean**	**SD**	**Min**.	**P_25_**	**P_50_**	**P_75_**	**Max**.
No. of cases	30	125	0	0	0	4	2,389
Tmean (°C)	13.29	10.93	−23.21	6.07	14.78	22.11	31.96
RF (mm)	77.63	82.63	0.00	15.51	50.76	115.01	982.00
RH (%)	66.30	13.25	27.43	57.29	69.13	77.14	89.33
SH (h)	174.68	57.81	11.43	135.68	178.86	217.09	325.45

SD, standard deviation; Min, minimum; P_25_, 25th percentile; P_50_, median; P_75_, 75th percentile; Max, maximum.

### Relationship between weather condition and scrub typhus

**Figure 5:**
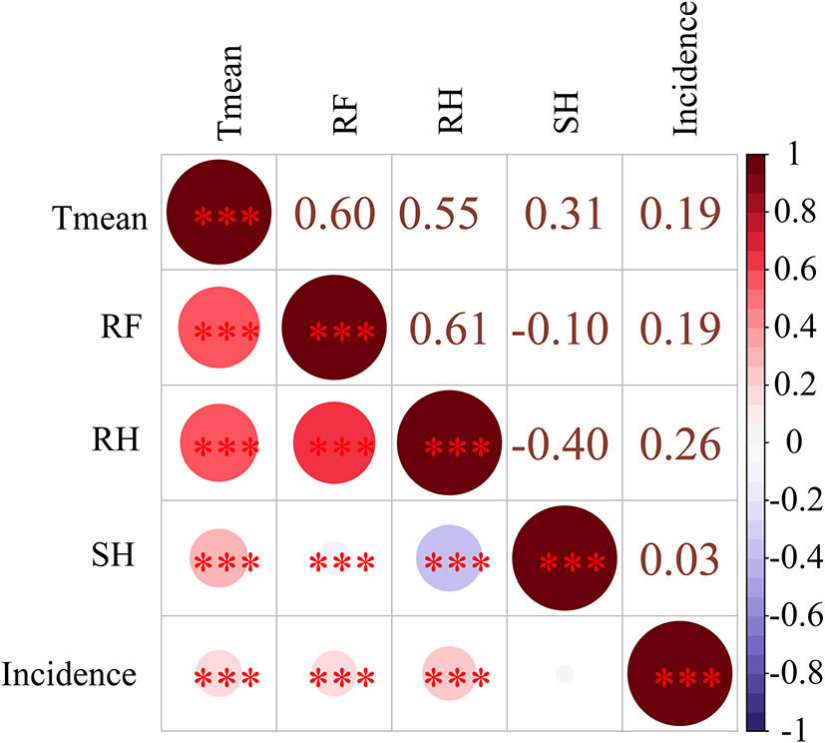
The Pearson's correlation between four weather conditions and scrub typhus incidence in China from 2006 to 2018. ***means ≤0.001; **means 0.01 ≥ *p* > 0.001; *means 0.05 ≥ *p* > 0.01. Mean temperature (Tmean), rainfall (RF), sunshine hours (SH), and relative humidity (RH).

*Pearson's* correlation analysis of ten high incidence provinces revealed that the incidence of scrub typhus was significantly positively correlated with three weather conditions, including monthly Tmean (*r* = 0.19), RF (*r* = 0.19) and RH (*r* = 0.26) (Figure 5).

**Figure 6:**
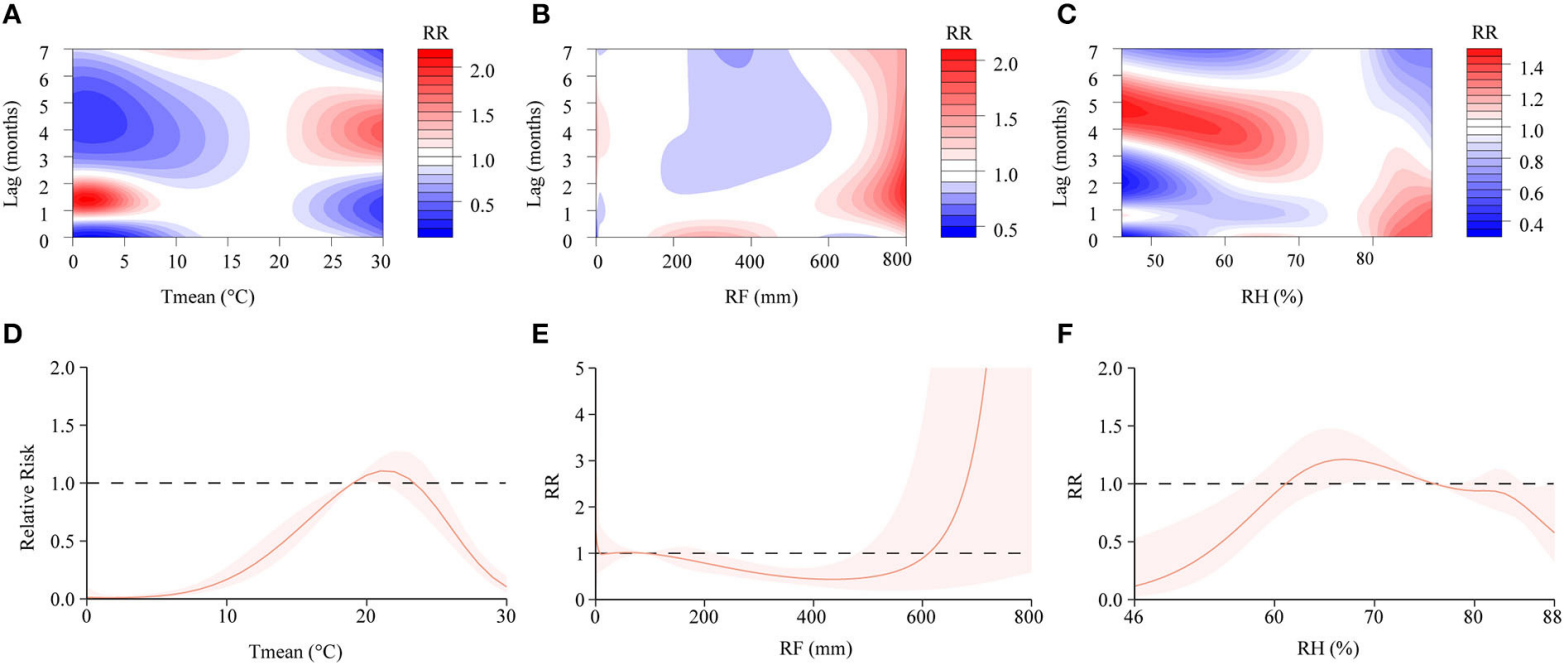
Contour plots and cumulative exposure-response curves of exposure-response relationships between scrub typhus incidence and three meteorological conditions in the single-variable model. **(A–C)** The y-axis represents the lag period from 0 to 7 months. The x-axis represents the range of observations for each variable. The color gradient represents relative risk (RR). Red represents RR > 1, blue represents RR < 1, and white represents no difference when RR =1. **(D–F)** The y-axis represents the RR. The x-axis represents the range of observations for each variable. Mean temperature (Tmean), rainfall (RF), and relative humidity (RH).

**Figure 7:**
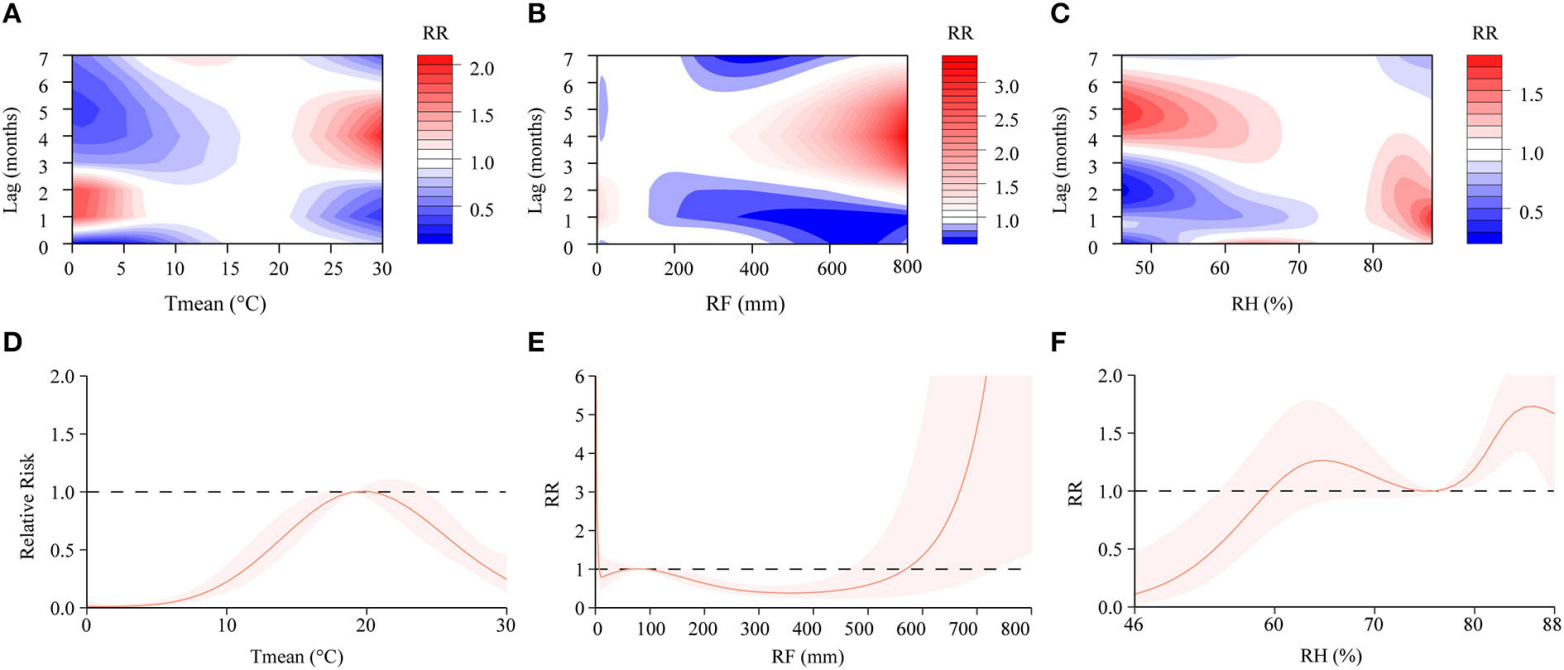
Contour plots and cumulative exposure-response curves of exposure-response relationships between scrub typhus incidence and three meteorological conditions in the multiple-variable model. **(A–C)** The y-axis represents the lag period from 0 to 7 months. The x-axis represents the range of observations for each variable. The color gradient represents relative risk (RR). Red represents RR > 1, blue represents RR < 1, and white represents no difference when RR = 1. **(D–F)** The y-axis represents the RR. The x-axis represents the range of observations for each variable. Mean temperature (Tmean), rainfall (RF), and relative humidity (RH).

In the DLNM, exposure-response curves showed a substantial linear association between scrub typhus and meteorological conditions with a lag of 0–7 months. In the single-variable models, three meteorological conditions were associated with scrub typhus incidence (Figures 6A–C; Supplementary Figure 5). The RR of Tmean, RH and RF were 0.15–2.16, 0.31–1.49 and 0.44–2.03, respectively. In the multiple-variable models, the RR was 0.15–2.03 for Tmean, 0.27–1.71 for RH, and 0.60–3.33 for RF (Figures 7A–C; Supplementary Figure 6). The maximum RR for Tmean at 0°C was 2.28 (95% CI: 0.95–5.43) with a lag of 1.4 months. The maximum RR for RH at 46% was 1.71 (95% CI: 1.39–2.09) with a lag of 4.8 months. The maximum RR for RF at 800 mm was 3.33 (95% CI: 1.89–5.86) with a lag of 4 months.

We used the median of weather factor as a reference and calculated the relative variable in different lag months (Table 3). In lag 3, Tmean was most significant at 30°C (RR = 1.47, 95% CI: 1.34–1.63). RH was most significant at 84% (RR = 1.24, 95% CI: 1.17–1.31). RF was most significant at 800 mm (RR = 2.63, 95% CI: 1.58–4.37).

**Table 3:** Maximum RR values and meteorological parameters under different lag months.

**Lag months**	**Tmean (°C)**	**RR (95% CI)**	**RH (%)**	**RR (95% CI)**	**RF (mm)**	**RR (95% CI)**
0	21	1.01 (0.96–1.06)	64	1.36 (1.17–1.57)	270	1.12 (0.99–1.26)
1	0	1.77 (0.80–3.93)	88	1.70 (1.46–1.98)	0	2.22 (1.09–4.54)
2	0	1.76 (0.89–3.50)	85	1.35 (1.25–1.44)	0	2.05 (1.08–3.89)
3	30	1.47 (1.34–1.63)	84	1.24 (1.17–1.31)	800	2.63 (1.58–4.37)
4	30	2.03 (1.78–2.31)	46	1.38 (1.13–1.70)	800	3.33 (1.89–5.86)
5	30	1.62 (1.42–1.84)	46	1.71 (1.41–2.07)	800	2.74 (1.61–4.67)
6	23	1.01 (0.95–1.07)	46	1.35 (1.19–1.54)	800	1.69 (0.89–3.19)
7	12	1.19 (1.07–1.32)	73	1.01 (0.99–1.04)	0	1.25 (1.00–1.57)

Tmean, mean temperature; RH, relative humidity; RF, rainfall.

### Cumulative risks with a lag of 0–7 months

We found that the cumulative risks of meteorological factors with a lag of 0–7 months were associated with scrub typhus incidence. In the single-variable models, the Tmean at 19–23°C, the RH at 62–76%, and the RF at 0–8 mm, 29–90 mm and 612–800 mm were all positively associated with scrub typhus risk (Figures 6D–F). In the multiple-variable models, the Tmean at 19–20°C, the RH at 60–74% and 76–88%, and the RF at 0–7 mm, 66–90 mm and 570–800 mm were all positively associated with scrub typhus risk (Figures 7D–F).

## Discussion

China is one of the regions where the incidence of scrub typhus is relatively obvious, with cases mainly distributed in Yunnan, Guangdong, Hainan, Fujian and other regions south of the Yangtze River. Since 1986, the epidemic focus has begun to appear in the area north of the Yangtze River. The distribution of scrub typhus incidences varied among provinces and cities in China, and the number of cases was uneven, which may be caused by socioeconomic factors such as population density and the difference in land use types or natural environments such as temperature, precipitation, and vegetation. This study conducted an epidemiological analysis on the top ten provinces with scrub typhus incidence, and explored the relationship between meteorological factors and the incidence through the DLNM.

This study revealed that scrub typhus mainly existed in the economically underdeveloped areas of southern China, such as Yunnan, Guangxi, and Jiangxi, which led us to doubt whether economic and social factors would affect scrub typhus (18). Shah et al. found that the increase in agricultural land area and the cultivation of oil palm and rubber in Southeast Asia reduced the natural forest and provided more favorable conditions for the breeding and life cycle of chigger mites, leading to an increase incidence of scrub typhus (19). Ranjan and Prakash also found that changes in land use, land cover types and urbanization led to an increase in rodent populations, and human activities such as hiking and camping increased human exposure opportunities to chiggers, which caused a surge in scrub typhus in India (20). By constructing a multiple regression model, Wardrop et al. identified scrub typhus was positively correlated with the proportion of agricultural population and land coverage, and was negatively correlated with per capita annual income. Interestingly, Li et al. also discovered a positive correlation between GDP and scrub typhus incidence in Guangdong, the major fruit producing area in southern China. Guangdong had more exposure opportunities due to more human planting economic behavior (21). It is currently known that some socioeconomic factors play an important role in the development of scrub typhus. How the level of socioeconomic development affects the incidence of scrub typhus remains to be further explored.

Our results showed that the high-incidence seasons of scrub typhus in northern and southern China were different, which may be related to the difference in the chiggers' life cycle. The main vectors in the southern region such as Guangdong, Fujian, Yunnan were *L.deliense* with a 3-month life cycle (22, 23). The main vectors in northern China such as Shandong, Jiangsu, Anhui were *L.scutellare*, which involved a long life history of more than 9 months (24, 25). It was worth noting that, as the main vector of scrub typhus in the southern summer, *L.deliense* peaked in June-July in coastal Fujian and declined in September, while in Guangdong it peaked in June-July and September-October. The incidence of scrub typhus varies from place to place, suggesting that public health departments need to adapt to local conditions and carry out targeted prevention efforts in different regions (26).

Previous studies showed that temperature was an important factor affecting the incidence of scrub typhus, which was consistent with our discovery (10, 27). The analysis found that the lag effect was obvious when the monthly mean temperature was higher, and the risk of disease increased with temperature. Scrub typhus was found to be positively correlated with higher temperature, humidity and precipitation in Laos (27). Through *Poisson* regression model and distributed lag non-linear model (DLNM), Through *Poisson* regression model and DLNM, Wei et al. found that the risk of scrub typhus increased by 3.8% for every 1°C increase in monthly surface temperature; the risk of scrub typhus increased by 5.3% for every 1°C increase in daily temperature, with a lag of 7 weeks (10). Similar results were seen in northern and northeastern Thailand, where scrub typhus incidence was associated with a 2-month lag of mean temperature (R = 0.55) and a 1-month lag of precipitation (R = 0.46) (28).

The life cycle and reproduction characteristics of chiggers may be one reason. Higher temperature and rainfall were more suitable for the growth and reproduction of chigger larvae (29), and the temperature range can affect the abundance and distribution of chiggers (12). Interestingly, Xu et al. found an intricate relationship between Scrub typhus growth and temperature. For example, *L.deliense* was the most representative chigger vector in southern China and Southeast Asia. When the temperature reached 23±1°C, the hatching rate of *L.deliense* was the highest (30). Too high or too low temperatures would reduce the hatching rate. This was consistent with our results that when the temperature was <10°C or more than 40°C, the larvae of the *L.deliense* could not develop. The difference in the incidence of scrub typhus under different temperature may be related to the non-linear relationship between different chigger species, host population dynamics and natural environmental factors (31, 32).

The cumulative risk of rainfall in this study showed that the monthly average rainfall involved first increase, then decreased, and then increased sharply, indicating that suitable rainfall was beneficial to the life activities of the hosts (rodents and chigger larvae). Currently, many vector-borne infectious diseases (HFRS, dengue fever, and bacillary dysentery, etc.,) are found to be positively correlated with precipitation, and disease transmission may be affected by the growth and development of pathogens and vectors/hosts (33–36). When rainfall exceeds a certain range, the sharp increase of scrub typhus may be linked to the heavy rainfall that is beneficial to the survival and reproduction of the host and human activities. Chigger larvae migrate with flood caused by heavy rainfall, increasing their range and exposure to humans (37, 38).

The lag analysis of humidity in this study displayed the different results between the single-variable and multiple-variable models. Results of single-variable model showed that high humidity could increase the scrub typhus risk, but when the humidity exceeded the critical value, it became an inhibitory effect on the risk. High humidity was also thought to increase the risk of scrub typhus. Yang et al. found that every 1% increase in monthly relative humidity in the first two months was associated with a 12.6% increase in monthly cases, which was consistent with our results (39). Moreover, humidity was thought to be related to the survival rate and egg-laying ability of chiggers. As humidity decreased, the number or activity of chiggers would decrease, and the spawning rate of adult chiggers would start to decline or even stop (40). As the relative humidity increased, the hatching rate of chigger larvae began to rise, and the larvae always survived and reproduced well (10, 41, 42). However, Lu et al. found that the results were not statistically significantly different when the relative humidity was more than 72%, which was consistent with the results of our single-variable study, but the reason was unclear. The incidence of scrub typhus under different humidity should be further explored (43).

In conclusion, (relatively) high temperature, high humidity and sufficient rainfall were all associated with the incidence of scrub typhus, and there was a certain cumulative lag effect. Changes in natural and socioeconomic factors would have a huge impact on the habitat of rodents and chiggers, thereby affecting the population distribution of rodents and chiggers. Therefore, in areas with high incidence of scrub typhus such as Yunnan and Guangdong, targeted prevention and control measures should be carried out during the epidemic season, and health education and personal protection awareness should be strengthened for vulnerable groups such as the middle-aged and elderly, farmers and diaspora children. The health department should strengthen local scrub typhus monitoring to detect and treat in time. At the same time, further studies should be conducted to find out the key risk factors affecting the rise of scrub typhus incidence, so as to provide a theoretical basis for scientific and effective prevention and control of scrub typhus.

This study has several limitations. First, we lack the epidemiological data of scrub typhus after 2018, and we cannot analyze the prevalence of scrub typhus in recent years. Second, we cannot extrapolate the short-term impact of meteorological factors on scrub typhus from monthly data. Third, data on some socioeconomic factors, including GDP, urbanization level, etc., are not collected. In future, the impact of meteorological factors and socioeconomic environment on scrub typhus needs to be comprehensively considered.

## Data availability statement

The original contributions presented in the study are included in the article/Supplementary material, further inquiries can be directed to the corresponding author.

## Ethics statement

This study obtained the permission of the Ethics Committee of the Nanjing Bioengineering (Gene) Technology Center for Medicines (Ethics Committee Batch No: 2018001003). Our study did not involve any private and personal information about scrub typhus cases. The data was anonymous.

## Author contributions

YL and LoZ: data curation, conception, software, and writing—original draft. HL, YQ, NY, LA, and CZ: conception, methodology, and software. LoZ and JW: data curation and software. LoZ: supervision. YL and WT: data collection, visualization, and writing—review and editing. All authors contributed to the article and approved the submitted version.

## Funding

This study was supported by the Jiangsu Social Development Project (BE2017620) and National Natural Science Foundation of China (U1602223).

## Conflict of interest

The authors declare that the research was conducted in the absence of any commercial or financial relationships that could be construed as a potential conflict of interest.

## Publisher's note

All claims expressed in this article are solely those of the authors and do not necessarily represent those of their affiliated organizations, or those of the publisher, the editors and the reviewers. Any product that may be evaluated in this article, or claim that may be made by its manufacturer, is not guaranteed or endorsed by the publisher.
