# Supplementary material for: How meteorological factors impacting on scrub typhus incidences in the main epidemic areas of 10 provinces, China, 2006–2018
Source: Front Public Health. 2022 Oct 19;10:992555. doi: 10.3389/fpubh.2022.992555 (PMC9628745; doi:10.3389/fpubh.2022.992555)
Supplement: Supplementary file 1 [file Data_Sheet_1.PDF]

Supplementary Information

Analysis of the effect of meteorological factors on scrub typhus in 10 provinces, China, 2006-2018

Supplementary Figure 1. Geographical location of meteorological monitoring stations in China.

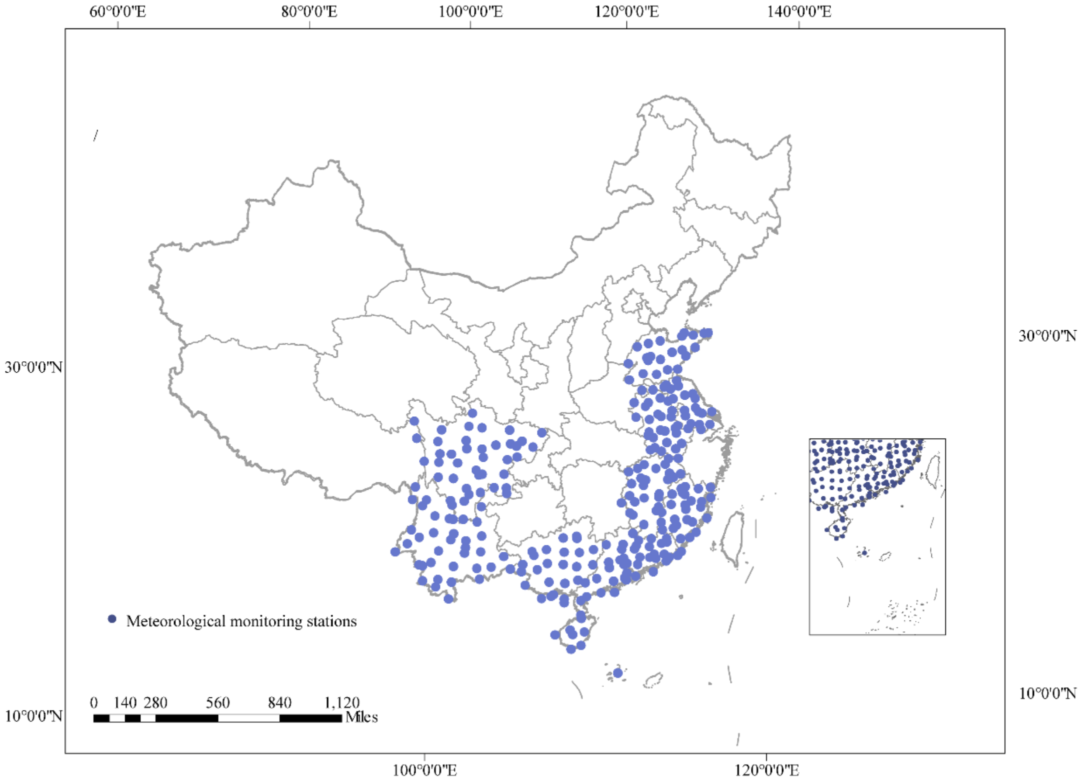

meteorological monitoring stations (N=272).

Supplementary Figure 2. Trends in incidence of scrub typhus and joinpoints in 10 provinces, China, 2006-2018.

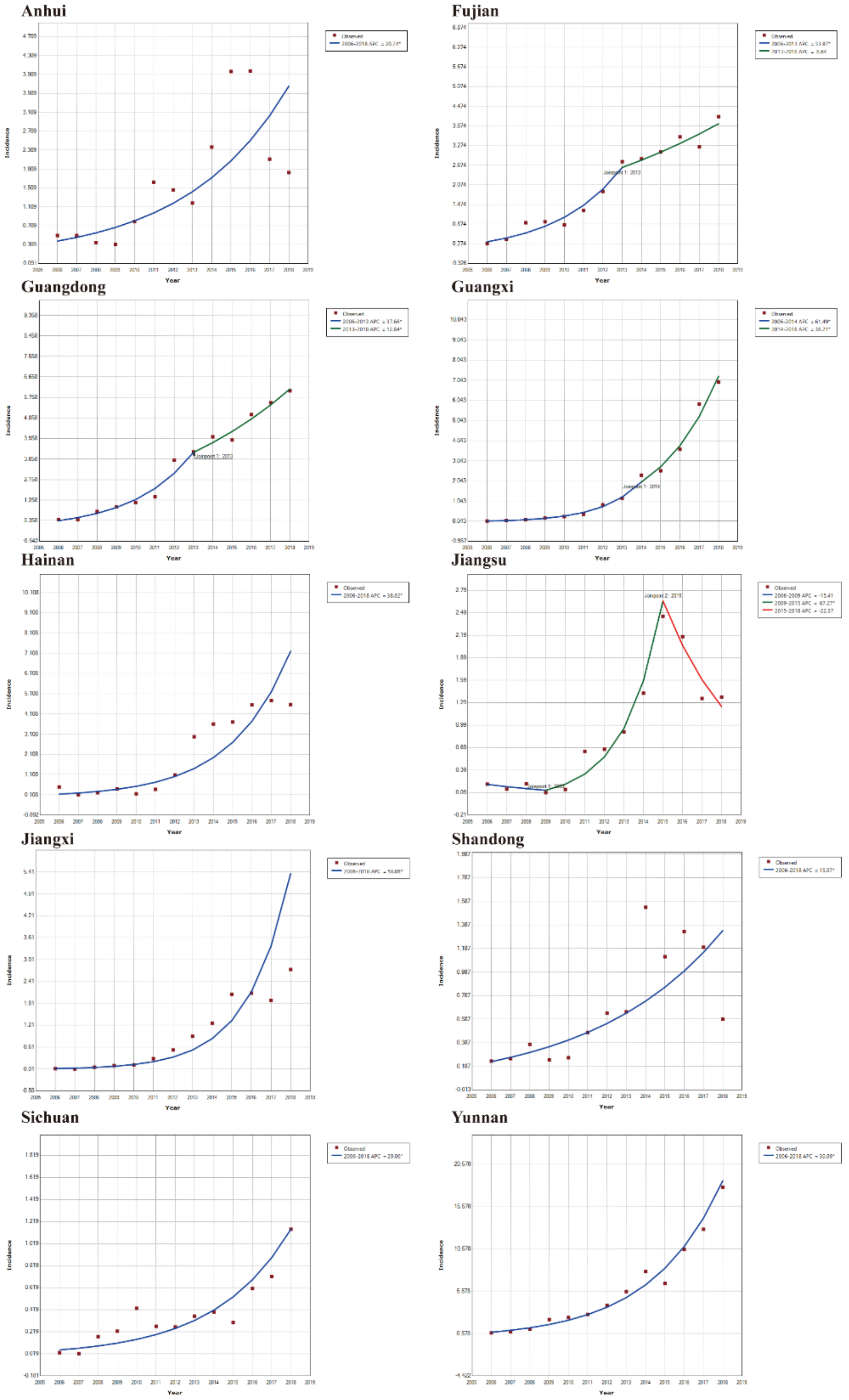

*Statistically significant trends. APC = Annual Percentage Change.

Supplementary Figure 3. Seasonal pattern of scrub typhus notifications in 10 provinces, China, 2006-2018.

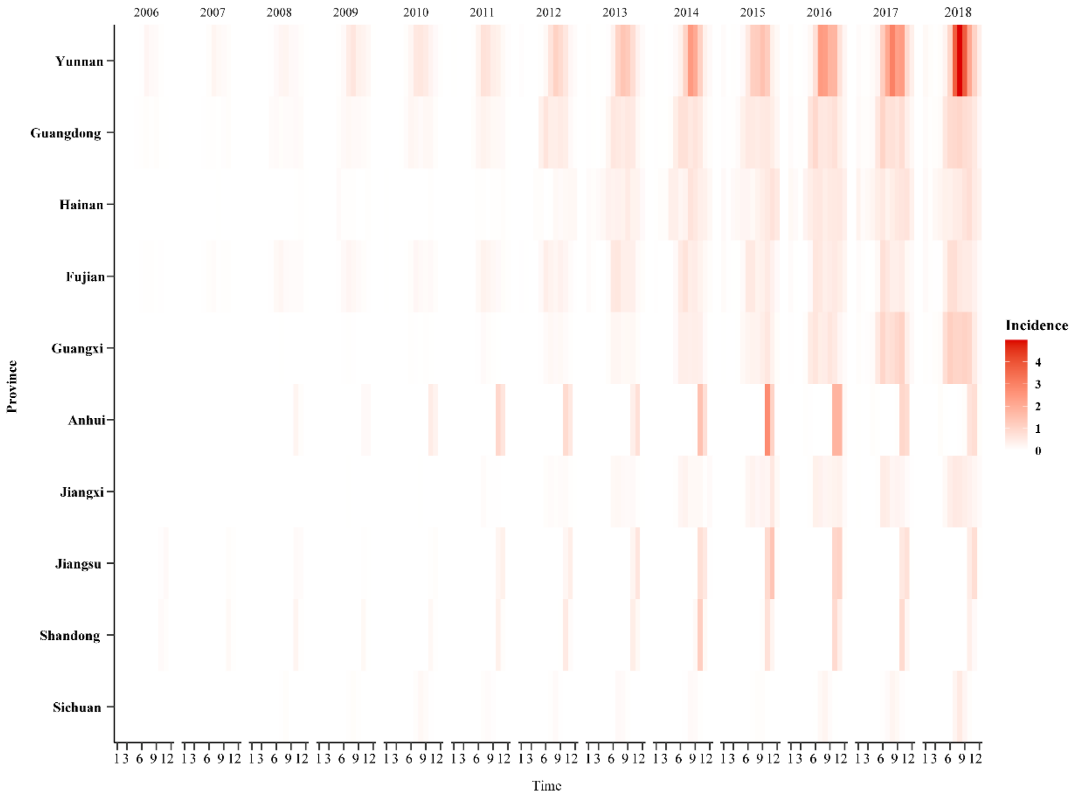

Supplementary Figure 4. The boxplots of four meteorological conditions and the number of scrub typhus cases in four seasons from 2006 to 2018 in 10 provinces, China.

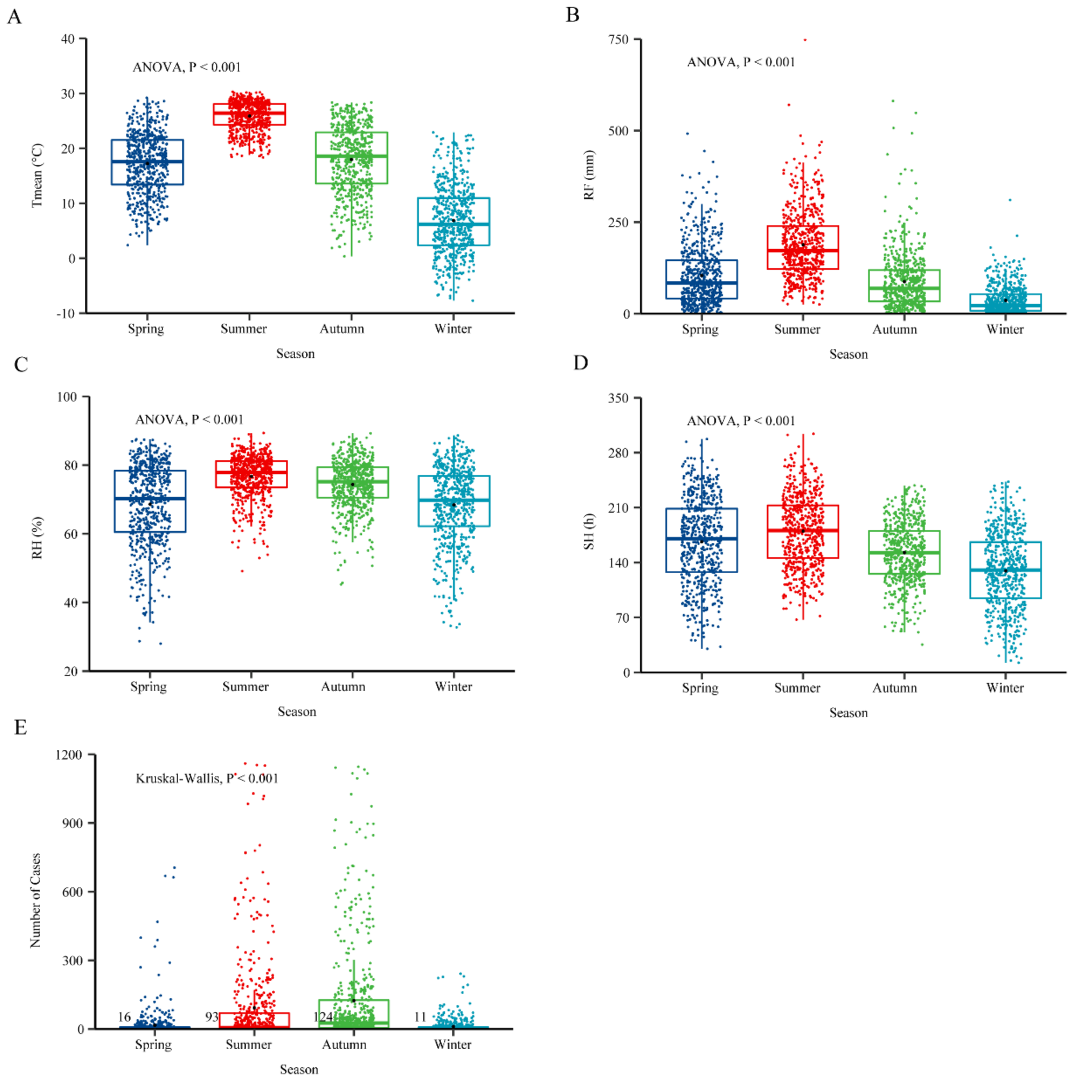

A-D Seasonal patterns of weather conditions. **E** Seasonal patterns of scrub typhus. The analysis of variance (ANOVA) test was applied to test whether the values between the four seasons were statistically significant. The *Kruskal-Wallis* test was used to detect the cases of scrub typhus in four seasons, spring (March-May), summer (June-August), autumn (September-November) and winter (December-February).

mean temperature (Tmean), rainfall (RF), sunshine hours (SH), and relative humidity (RH).

Supplementary Figure 5. 3-D plot of scrub typhus incidence by three meteorological factors with lag 0-7 months from 2006 to 2018 in the single-variable model

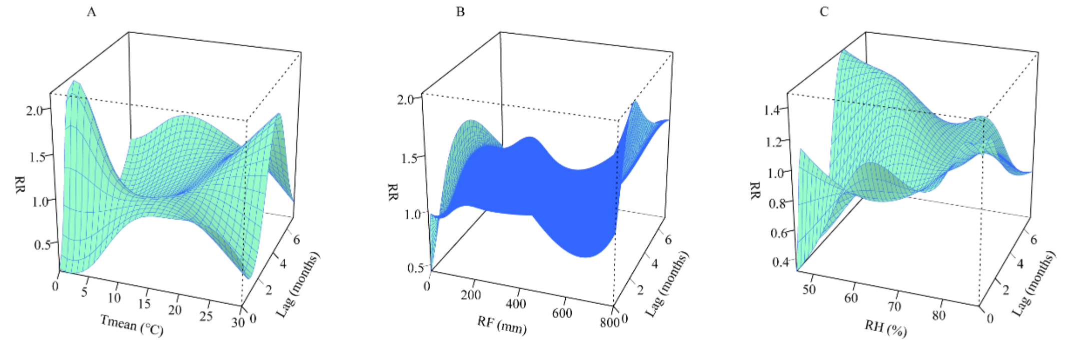

mean temperature (Tmean), rainfall (RF), and relative humidity (RH).

Supplementary Figure 6. 3-D plot of scrub typhus incidence by three meteorological factors with lag 0-7 months from 2006 to 2018 in the multiple-variable model

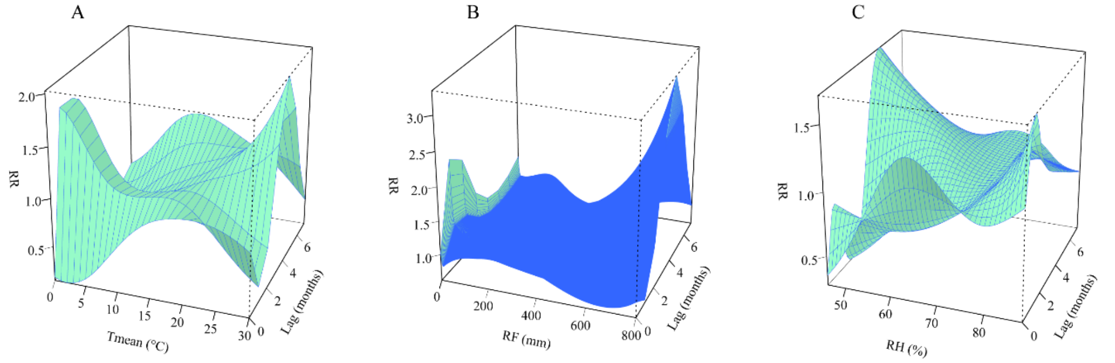

mean temperature (Tmean), rainfall (RF), and relative humidity (RH).

Table S1. Descriptive statistics for monthly scrub typhus cases and weather conditions in 10 provinces, 2006-2018 (n=1560)

Variables	Mean	SD	Min.	P_{25}	P_{50}	P_{75}	Max.
**No. of cases**	88	208	0	1	10	62	2389
**Tmean (°C)**	18.08	7.94	-4.44	12.42	19.39	24.89	30.30
**RF (mm)**	115.96	102.53	0.52	34.80	90.10	168.02	982.00
**RH (%)**	74.19	7.96	45.12	69.53	76.02	80.28	88.97
**SH (h)**	155.94	49.09	12.35	123.69	155.68	190.08	297.32

mean temperature (Tmean), rainfall (RF), sunshine hours (SH), and relative humidity (RH).
